# Intramedullary nailing versus plate compound osteosynthesis in subtrochanteric and diaphyseal pathologic femoral fractures: a retrospective cohort study

**DOI:** 10.1007/s00590-023-03599-7

**Published:** 2023-05-29

**Authors:** Sebastian Koob, Milena Maria Plöger, Johanna Sophie Schmolling, Ramona Pia Lehmann, Dana Alex, Hendrik Kohlhof

**Affiliations:** 1 Department of Orthopaedics and Trauma Surgery, University of Bonn, Venusberg Campus 1, 53127 Bonn, Germany; 2 Department of Emergency, Hand and Orthopaedic Surgery, St. Antonius Hospital, Cologne, Germany

**Keywords:** Proximal femoral metastasis, Skeletal metastases, Metastatic disease, Palliative surgery, Skeletal-related events, Compound osteosynthesis

## Abstract

**Purpose:**

Pathologic fractures of the extremities due to carcinoma metastases require individual and patient prognosis-related stabilization procedures. Quick remobilization of the patient to restore the quality of life is of high importance, especially in the case of subtrochanteric and diaphyseal femoral fractures. In our retrospective cohort study, we evaluated intraoperative blood loss, length of operation, complication rate, and regain of lower extremity function in plate compound osteosynthesis (PCO) versus intramedullary nailing (IM) for subtrochanteric and diaphyseal pathologic fractures of the femur.

**Methods:**

Between January 2010 and July 2021, we retrospectively reviewed 49 patients who were treated at our institution for pathologic fractures of the subtrochanteric and diaphyseal femurs for group differences in terms of blood loss, length of operation, implant survival, and Musculoskeletal Tumor Society (MSTS) score.

**Results:**

We included 49 stabilization procedures of the lower extremity due to pathologic fractures of the proximal or diaphyseal femur, with a mean follow-up of 17.7 months. IM (*n* = 29) had a significantly shorter operation time than PCO (*n* = 20) (112.4 ± 9.4 and 163.3 ± 15.96 min, respectively). We did not detect any significant differences in terms of blood loss, complication rate, implant survival, or MSTS score.

**Conclusion:**

Based on our data, pathologic subtrochanteric and diaphyseal fractures of the femur can be stabilized with IM, which has a shorter operation time than PCO, but the complication rate, implant survival, and blood loss remain unaffected.

## Purpose

Because of improved oncologic therapeutic options and prolonged survival times of cancer patients, stabilization of impending and complete pathologic fractures of the extremities is gaining importance. Skeletal-related events due to metastatic disease have an increasing prevalence, and surgical procedures are most often necessary to restore skeletal stability, relieve pain, enable immediate full weight bearing, and thus regain quality of life. Since wide resection of the lesion shows no benefit to survival in multi-metastatic disease, osteosynthesis techniques are favorable. In cases of metastases of the proximal and diaphyseal femur, intramedullary nailing (IM) and plate compound osteosynthesis (PCO) are valid options of treatment if the native joint is to be preserved [1]. IM is predominantly used in radiosensitive and chemosensitive metastases and offers fixation of the entire long bone with small dissection, whereas open reduction and internal fixation (ORIF) using PCO provides better visualization and allows curettage and resection when tumor debulking is necessary. Especially in non-radiosensitive metastases, e.g., renal cell carcinoma, tumor debulking is recommended. Nonetheless, ORIF shows a higher rate of revision surgery than IM or prosthetic replacement, most often due to failure of fixation [2]. The current literature is limited and inconclusive as to which procedure has faster duration and remobilization and has higher implant survival and functionality due to the high rate of published cohorts of IM [3, 4], and outcome analyses of PCO in cases of metastatic fractures of the subtrochanteric and diaphyseal femurs are scarce. Furthermore, large multicenter cohorts do not include a high number of PCO [2, 5, 6]. The aim of our retrospective analysis was to answer the following questions. Is there a difference in intraoperative blood loss and operation time between IM and PCO? Does IM have a lower complication rate and higher implant survival rate than PCO?

## Materials and methods

The surgical stabilization using a compound plate osteosynthesis or intramedullary nailing in 49 patients with complete subtrochanteric and diaphyseal pathologic femoral fractures due to carcinoma metastases between January 2010 and July 2021 at our institution was retrospectively reviewed. Two groups were established according to stabilization: PCO and IM. The type of osteosynthesis in each patient was determined based on implant availability and surgeon’s preference at the time of surgery. Surgeries were performed by five skilled surgeons (> 6 years of experience) of the tumor orthopedic department at our institution. Demographic information, types of operation, operation time, intraoperative blood loss, and Musculoskeletal Tumor Society (MSTS) score [7] were retrieved from patient charts and intraoperative documentation. To obtain a more accurate value for intraoperative blood loss, a mean blood content of 50 and 5 mL was estimated for consumption of abdominal linen and compresses, respectively. All patients had multiple osseous metastases, either visceral or lymphatic, or both. The mean follow-up was 17.7 months (range 0–80 months). Of the 49 cases, PCO was performed in 20 patients (40.8%) and IM in 29 patients (59.2%). GraphPad Prism version 9.3.1 for Windows (GraphPad Software, San Diego, CA, USA; www.graphpad.com) was used for the calculation of descriptive statistics. The Welch’s t test and Fisher’s exact test were used for group comparisons. Kaplan–Meier curves were used for survival analysis.

## Results

**Fig. 1:**
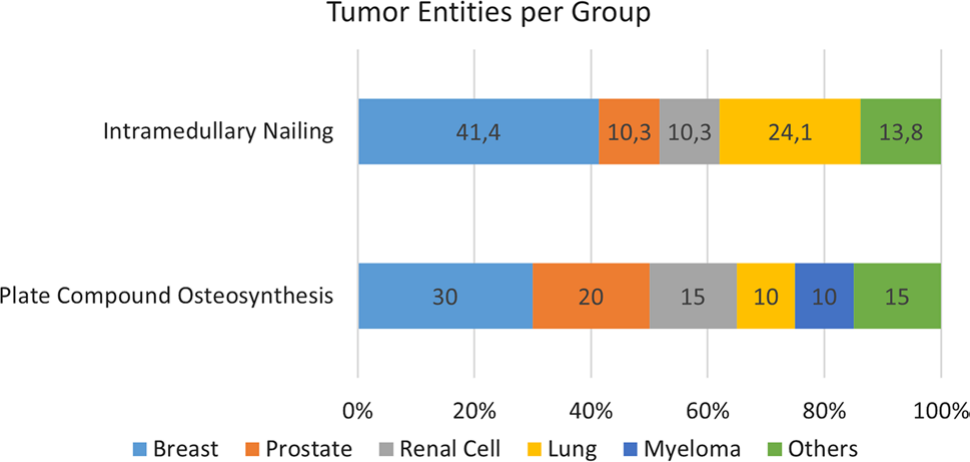
Tumor entities per group

In this retrospective study, we included 49 patients who underwent stabilization procedures of the subtrochanteric and diaphyseal femurs due to carcinoma metastases. In general, there were no important differences between the groups in terms of age (PCO group, 56.9 ± 14.73 years; IM group, 62.7 ± 12.1 years; *p* = 0.1360), sex (PCO group, 10 males and 10 females; IM group, 10 males and 19 females; *p* = 0.3773, Fisher’s exact test) and cancer stage. All the patients had multiple organ and bone metastases. Figure 1 shows the underlying malignancy of the tumor. The median patient survival was 10 months in the PCO group and 6 months in the IM group. This difference was not statistically significant (*p* = 0.2983). After 6 months, 68.4% and 44.8% of the patients in the PCO and IM groups were still alive, respectively. After a year, 42.1% and 34.5% of the patients in the PCO and IM groups were still alive, respectively.

**Fig. 2:**
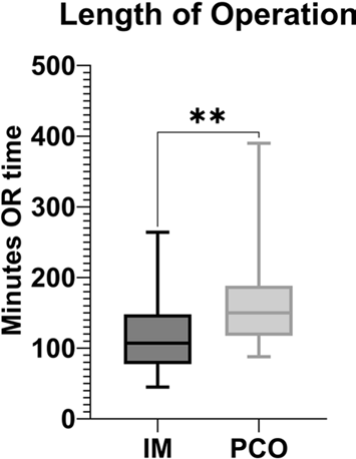
Length of operation

The mean blood loss did not differ between the PCO and IM groups (723.3 ± 336.1 versus 628.6 ± 249.1 mL; *p* = 0.8226), whereas the operation time was longer in the PCO group than in the IM group (163.3 ± 15.96 versus 112.4 ± 9.4 min; mean difference [MD], 50.92 ± 18.5 min; 95% confidence interval [CI], 13.22–88.62 min; *p* = 0.0097) (see Fig. 2).

**Fig. 3:**
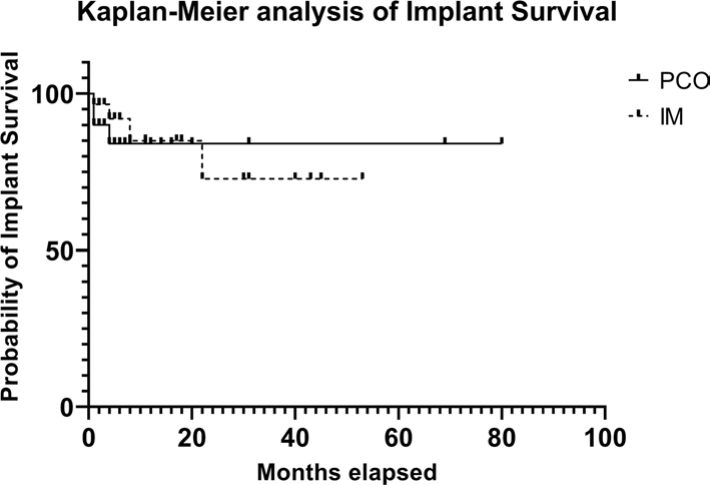
Kaplan–Meier analysis of implant survival

We detected a higher rate of complications in the PCO group (25%, *n* = 5, which includes 3 long-term plate loosening due to metastatic progression and conversion into modular tumor prosthesis and 2 postoperative wound infections with wound revision surgery and implant retention) than the IM group (17.24%, *n* = 5, which includes 1 wound infection and consecutive explantation of the implant due to deep infection, 3 conversions into endoprosthesis due to metastatic progression, and 1 femoral neck blade exchange due to articular perforation). This difference was not statistically significant (*p* = 0.7199, two-sided Fisher’s exact test). We did not detect a statistically significant difference in implant survival between the two groups (Kaplan–Meier survival analysis; Fig. 3). In three cases (15%) in the PCO group, the implant did not prolong patient survival. In the IM group, the implant did not survive the patient in 4 cases (13.8%).

We observed a higher preoperative MSTS score in the PCO group than that in the IM group (8.550 versus 4.138 points; MD, 4.412 ± 1.789 points; 95% CI, 0.7541–8.070 points; *p* = 0.0198). There was no difference in the median postoperative improvement in the MSTS score between the two groups (PCO group, 12.5 points; IM group, 11 points). The resulting postoperative MSTS score was higher in the PCO group than that in the IM group (20.2 versus 14.31 points; MD, 5.890 ± 1.740 points; 95% CI, 2.371–9.408 points; *p* = 0.0016).

Unfortunately, although full weight bearing was intended for every treated patient, none of the treated patients achieved full weight bearing at the time of discharge from our clinic, and only 4 (20%) and 5 (17.24%) patients in the PCO and IM groups, respectively, achieved a documented full weight bearing during follow-up. The difference was not statistically significant (*p* > 0.9999, two-sided Fisher’s exact test).

## Discussion

When dealing with pathologic fractures of the subtrochanteric and diaphyseal femur in a multi-metastatic situation and when wide excision is not mandatory because of the lack of prognostic influence, less invasive procedures such as IM and PCO are favorable options. Owing to the lack of sufficient data, the decision between these two procedures is often made based on the surgeon’s preference and implant availability. The literature shows a tendency toward IM, as it offers minimal invasive stabilization of the complete femur [8]. Meanwhile, PCO is accompanied by a better exposition of the local bone lesion for curettage, but it often results in longer incision lines and a significant risk of secondary vascular complications [9]. We conducted our study to evaluate group differences between PCO and IM in the stabilization of subtrochanteric and diaphyseal pathologic fractures in terms of blood loss, operation time, complication rate, and functionality. We did not find differences in blood loss, complication rate, or gain in lower extremity function, but we observed that the PCO group had a longer operation time.

The most important limitation of our study is based on its retrospective nature and lack of blinding between the two study groups. Furthermore, the decision between the two stabilization options was obviously not made according to a certain study protocol, but rather on the preference of the surgeon and implant availability. Therefore, this study also served the purpose of evaluating our efforts and providing a statistical idea for future decisions between these two stabilization methods and, consequently, the treatment algorithm at our clinic. The cohort of patients seems to be small, but the number of patients treated with PCO is relatively higher than that in the current literature. Even multicenter cohorts do not accumulate large numbers of PCO stabilization (Meynard et al. [5], 309 patients included, with 12 PCO; Gruber et al. [6], 109 patients included, with 20 PCO [6]; Janssen et al. [2],417 patients included, with 24 PCO), which might be explained by the surgeon’s preference for IM or endoprosthetic reconstruction, as shown in an MSTS survey in 2013 [10]. Another limitation lies in the relatively short follow-up of 17.7 months, which was mainly caused by limited survival due to the oncologic situation in multi-metastatic patients, which was also observed in other studies using the same stabilization methods (Moon et al. [11], 13 months of follow-up; Meynard et al. [5], 9 months of follow-up; Janssen et al. [2] 4 months of follow-up). Therefore, implant survival during patient survival is of crucial interest since patients do not want to undergo revision surgery during their limited life span. We did not observe a difference in implant survival between the two groups, but 15% of the implants in the PCO group and 13.7% in the IM group did not match the patients’ survival. These implant failures were caused by deep implant infections and loosening due to metastatic progression. Unfortunately, the overall aim of reliable pathologic fracture stabilization in oncologic patients to ensure and preserve a limited quality of life during a limited life span was not achieved in these cases.

Our first key objective was to answer the question concerning differences in blood loss and operation time between the two groups, as fast and less invasive interventions should be preferred in oncologic patients. Surprisingly, our retrospective study showed no significant difference in intraoperative blood loss, even though PCO includes a wider exposition and curettage of the metastasis and bone. We found only one other study with a comparison PCO and IM in terms of blood loss. Janssen et al. reported a significant difference between blood loss in IM (200 mL) and PCO (275 mL) [2]. The study did not include information on how intraoperative blood loss was estimated. The higher blood loss observed in our cohort might be explained by the fact that we included the consumption of abdominal linen and compresses to achieve a more precise value (50 and 5 mL for abdominal linen and compresses, respectively).

The operation time was significantly shorter in the IM group than in the PCO group. Although Janssen et al. reported a longer duration for both types of stabilization than we did, they did not find a statistical difference [2]. Gao et al. reported an even shorter operation time for IM in metastatic proximal femoral stabilizations of 59.5 min, but they did not include PCO in their analysis [12]. The authors could not find sufficient data from other cohorts concerning operation time in PCO, but because of the less invasive nature of IM with small incisions, a shorter operation time is reasonable. From the authors’ point of view, a longer than usual operation time in IM is mainly caused by unsuccessful attempts at closed pathologic fracture reduction. In such cases, a direct open approach and plate fixation may be advantageous.

The second key objective of our study was to closely examine the complication rates. We observed a slightly favorable situation concerning overall complications for the IM, but it was not statistically significant (17.24% versus 25%; *p* = 0.7199). It was in the same range as other IM and PCO studies in metastatic situations [3, 6, 13], but it was higher than that in the large cohorts of Janssen et al. (IM, 5.3%; PCO, 13%) [2] and Meynard et al. (IM, 1.4%; PCO, 16%) [5]. The above-mentioned implant failure rate often leads to the question of whether direct endoprosthetic replacement, especially in situations involving the intertrochanteric area, should be preferred [14]. Steensma and Healey reported a complication rate for endoprosthetic replacement in metastatic situations of the proximal femur of 6.1% and an implant failure rate of 0.5% with a longer durability than IM or PCO [10]. However, complications occurred within 90 days (mainly dislocations) after the surgery.

The regaining of lower extremity functionality was almost equal in both groups and did not favor either of the stabilization types, but we detected a slightly higher preoperative and postoperative MSTS scores in the PCO group. Surprisingly, the probability of achieving full weight bearing during follow-up was low in both groups (PCO, 20%; IM, 17.24%; *p* > 0.99). Unfortunately, other studies did not provide information on the rate of re-achievement of full weight bearing in their patient cohorts. From the authors’ point of view, these low rates of full weight bearing are disturbing, and patients should be well informed of that prognosis prior to surgery to avoid false expectations. However, considering the specific situation of a multi-metastatic patient with a pathologic fracture, stabilization should be conducted as soon as possible with a reasonable length of surgery. Based on our data, IM offers an equal amount of functionality and complication rate as PCO with a shorter operation time. Compared with a modular endoprosthesis, the main benefit of the procedure lies in the preservation of the native joint without prosthesis-induced complications (dislocation and periprosthetic joint infection). The most feared complication we observed was implant failure due to metastatic progression, which led to implant failure in 7 (14.3%) of the 49 cases.

## Conclusion

Based on our data, IM and PCO offer a comparable rate of complications, intraoperative blood loss, and regain of functionality in cases of pathologic subtrochanteric and diaphyseal femoral fractures. IM showed a significantly shorter operation time and additionally enabled stabilization of the total femur in one intervention. We, therefore, consider it as the first choice in applicable situations. In cases where open reduction and curettage of the bone lesion are necessary, PCO is still a valid and useful option. In cases of prognostic survivorship over a year, endoprosthetic replacement should be primarily considered due to its long durability.

## Data Availability

Not applicable.

## Abbreviations

IM: Intramedullary nailing
MSTS: Musculoskeletal Tumor Society
ORIF: Open reduction and internal fixation
PCO: Plate compound osteosynthesis

## References

[1] Koob S, Kehrer M, Strauss A, Jacobs C, Wirtz DC, Schmolders J (2019). Bone metastases–pathophysiology, diagnostic testing and therapy (Part 2). Z Orthop Unfall.

[2] Janssen SJ, Kortlever JT, Ready JE, Raskin KA, Ferrone ML, Hornicek FJ, Lozano-Calderon SA, Schwab JH (2016). Complications after surgical management of proximal femoral metastasis: a retrospective study of 417 patients. J Am Acad Orthop Surg.

[3] Chafey DH, Lewis VO, Satcher RL, Moon BS, Lin PP (2018). Is a Cephalomedullary nail durable treatment for patients with metastatic peritrochanteric disease?. Clin Orthop Relat Res.

[4] Willeumier JJ, Kaynak M, van der Zwaal P, Meylaerts SAG, Mathijssen NMC, Jutte PC, Tsagozis P, Wedin R, van de Sande MAJ, Fiocco M, Dijkstra PDS (2018). What factors are associated with implant breakage and revision after intramedullary nailing for femoral metastases?. Clin Orthop Relat Res.

[5] Meynard P, Seguineau A, Laumonerie P, Fabre T, Foltran D, Niglis L, Descamps J, Bouthors C, Lebaron M, Szymanski C, Sailhan F, Bonnevialle P, MembersoftheSoF (2020). Surgical management of proximal femoral metastasis: fixation or hip replacement? A 309 case series. Orthop Traumatol Surg Res..

[6] Gruber G, Zacherl M, Leithner A, Giessauf C, Glehr M, Clar H, Windhager R (2009) Surgical treatment of pathologic fractures of the humerus and femur. Orthopade. 38 (4):324, 326–328, 330–324. 10.1007/s00132-008-1376-4 10.1007/s00132-008-1376-4 19296079

[7] Enneking WF, Dunham W, Gebhardt MC, Malawar M, Pritchard DJ (1993). A system for the functional evaluation of reconstructive procedures after surgical treatment of tumors of the musculoskeletal system. Clin Orthop Relat Res.

[8] Piccioli A, Rossi B, Scaramuzzo L, Spinelli MS, Yang Z, Maccauro G (2014). Intramedullary nailing for treatment of pathologic femoral fractures due to metastases. Injury.

[9] Neubauer T, Grechenig S, Leitner L, Auffarth A, Plecko M (2016). Vascular complications in plating of the proximal femur: review. Arch Orthop Trauma Surg.

[10] Steensma M, Healey JH (2013). Trends in the surgical treatment of pathologic proximal femur fractures among Musculoskeletal Tumor Society members. Clin Orthop Relat Res.

[11] Moon B, Lin P, Satcher R, Bird J, Lewis V (2015). Intramedullary nailing of femoral diaphyseal metastases: Is it necessary to protect the femoral neck?. Clin Orthop Relat Res.

[12] Gao H, Liu Z, Wang B, Guo A (2016). Clinical and functional comparison of endoprosthetic replacement with intramedullary nailing for treating proximal femur metastasis. Chin J Cancer Res.

[13] Mohamed-Haflah NH, Kassim Y, Zuchri I, Zulmi W (2017). Outcome of skeletal reconstructive surgery for metastatic bone tumours in the femur. Malays Orthop J.

[14] Wedin R, Bauer HC (2005). Surgical treatment of skeletal metastatic lesions of the proximal femur: endoprosthesis or reconstruction nail?. J Bone Joint Surg Br.

